# A study of socio-economic inequalities in self-reported oral and general health in South-East Norway

**DOI:** 10.1038/s41598-022-18055-5

**Published:** 2022-08-12

**Authors:** Heidi Lyshol, Liv Grøtvedt, Tone Natland Fagerhaug, Astrid J. Feuerherm, Gry Jakhelln, Abhijit Sen

**Affiliations:** 1 Department of Health and Inequality, Norwegian Institute of Public Health, Oslo, Norway; 2 Center for Oral Health Services and Research (TkMidt), Trondheim, Norway; 3 Unaffiliated, Skien, Vestfold and Telemark Norway; 4 Department of Public Health and Nursing, Faculty of Medicine and Health Sciences, Norwegian University of Science and Technology, Trondheim, Norway; 5 Present Address: Norwegian Association of Dental Hygienists, Oslo, Norway

**Keywords:** Risk factors, Diseases, Oral diseases

## Abstract

This study assesses the association between socioeconomic determinants and self-reported health using data from a regional Norwegian health survey. We included 9,068 participants ≥ 25 years. Survey data were linked to registry data on education and income. Self-reported oral and general health were separately assessed and categorized into ‘good’/‘poor’. Exposures were educational level, personal income, and economic security. Prevalence ratios (PR) were computed to assess the associations between socioeconomic determinants and self-reported health using Poisson regression models. Participants with low education or income had poorer oral and general health than those with more education or higher income. Comparing the highest and lowest education levels, adjusted PRs for poor oral and general health were 1.27 (95%CI, 1.11–1.46) and 1.43 (95%CI, 1.29–1.59), respectively. Correspondingly, PRs for lowest income quintiles compared to highest quintile were 1.34 (95%CI, 1.17–1.55) and 2.10 (95%CI, 1.82–2.43). Low economic security was also significantly associated with poor oral and general health. There were socioeconomic gradients and positive linear trends between levels of education and income in relation to both outcomes (P-linear trends < 0.001). We found statistical evidence of effect modification by gender on the association between education and oral and general health, and by age group between income and oral health.

## Introduction

Oral health is an integral part of general health^1,2^, and a growing body of research has shown that both oral and general health vary with social determinants^2–6^. Good oral and general health are associated with higher socioeconomic status, and poor oral and general health with lower socioeconomic status^4,7,8^. These differences are often found to vary from top to bottom as gradients, with increasingly poor health with each lower category of the socioeconomic indicators^5,9^.

Despite being largely preventable, dental caries is the most common disease globally, with increasing prevalence in many countries^1,2^. In all countries, dentistry needs to be more integrated with primary care services, and more focused on promoting and maintaining oral health^2,9^. In 23 European countries, higher dental care coverage was found to be associated with smaller income inequalities in foregone dental care^10^. A recent Norwegian study found foregone dental visits due to financial reasons to be associated with poor self-assessed oral health, independent of age^11^. The importance of stratifying for age and gender when studying socioeconomic health inequalities has been shown in studies from Norway and Sweden, with consistent age and gender differences in oral and general health^8,11^.

Self-reported oral and general health have been suggested as reliable measures of health status. A study including data from 19 European countries found self-reported general health to be a valid and predictive measure for morbidity^12^. Another study from the USA demonstrated moderate to strong association of self-reported general health with mortality^13^.

Self-reported oral health has been found to be a valid estimate compared with clinical records, and with oral health-related quality of life^14–16^. Few studies from the Scandinavian countries have examined the association of social inequalities with oral health^11,16–19^ and general health^9,20–22^, and even fewer have looked at both outcomes^7,8^. Inequalities persist across most health outcomes, and there exists a clear social gradient^9,22^.

Important inequalities in the utilisation of health care services are found in Norway, which may contribute to sustaining inequalities in health outcomes. Self-rated general health was found to be a main predictor of health care utilisation^23^. Accordingly, perceived oral health has clearly been connected to utilization of dental services^24,25^. In Norway, poor self-reported oral health was associated with financial barriers for receiving dental health services^11^.

Norway is an interesting country when comparing oral and general health status, since the financing systems for dental health care and general health care differ^26^, and because social stratification seems to be less pronounced than elsewhere^27^. The present health care system in Norway covers all major expenses for somatic and mental health care. However, the great majority of adults aged over 20 are expected to cover their own dental care costs in full^26^, in contrast to the other Nordic countries, where the adult populations are eligible for at least partial reimbursement of dental related expenditures^28^.

Despite general good coverage of health care in Norway, the probability of an initial visit to a somatic specialist was higher among affluent and well-educated individuals^23^. Similar differences were found for the utilisation of dental services^29^. In an elongated country like Norway, structural inequalities, such as differences in travel distance across municipalities, may also affect the availability of healthcare services. These types of structural inequalities may not be independent of assignment to specific socioeconomic groups.

The objective of this study was to investigate the association of socioeconomic factors in relation to self-reported oral and general health. Due to the differences in the Norwegian financing systems for oral and general health care, we had the following hypotheses connected to the socioeconomic gradients: (i) the educational gradients for oral and general health would be more or less similar; (ii) the income gradient would be more pronounced for oral health than for general health; Further, we hypothesized that there might be effect modifications by age and gender to oral and general health status, consistent with earlier studies.

## Methods

From November 2015 to February 2016, a cross-sectional health interview survey was carried out in the 44 municipalities (clustered into 11 regions) of Vestfold, Aust-Agder, and Vest-Agder counties in Norway. As the main purpose of the survey was to support public health work at the local level, efforts were made to obtain a relatively large selection in each municipality, providing useful information at the administration level^30,31^. Around one-tenth of the Norwegian population live in the selected areas, and the population from which our sample was randomly selected was near the national average regarding education, individual income and age distribution. A dropout analysis was performed, showing that the selected population was representative for the adult population in the three counties^30^.

A two stage cluster sampling design was applied. A total of 22,700 adults aged 18 years or older living in the 44 varied municipalities (clustered into 11 regions) of three counties were randomly selected from the Norwegian Population Registry. We excluded individuals in prisons and nursing homes. The sample was proportional to the population in each region within the three counties. The overall response rate was 42.7% (9692 respondents)^30,31^. Information from the population registry (municipality, age, and sex) was delivered along with the questionnaire data.

Questionnaire data were linked to registries in Statistics Norway for information about education and personal income, using the personal identification number assigned to every resident of Norway. The educational scales are regularly harmonized with the International Standard Classification of Education (ISCED)^30,32^.

### Exposures

Highest achieved educational levels were grouped into three categories, primary school (≤ 10 years of schooling; ISCED categories 0–2), high school (11–13 years of schooling; ISCED categories 3–4), and higher education (any college or university education; ≥ 14 years of schooling; ISCED categories 5 +). Personal annual income after taxation was divided into quintiles, with Q1 as the lowest and Q5 as the highest income group. In 1000 NOK, Q1 =  < 216 (< 24,000 USD), Q2 = 216–277 (24–31,000 USD), Q3 = 277–335 (31–38,000 USD), Q4 = 335–417 (38–47,000 USD), and Q5 =  > 417 (> 47,000 USD).

Previous studies have included a question on economic security, i.e., whether the respondents could manage to raise a specific sum to cover an unexpected expense within 1 month^7,19^. We used a comparable question: “Could your household afford to pay an unexpected bill of 10,000 NOK (ca 1100 USD) without having to take out a loan or receive financial help?” Economic security was categorized as ‘yes’ (affording), and ‘no’ (not affording to pay).

### Outcome

Self-reported oral and general health were two different outcomes and were assessed by the questions “How do you rate your health in general?” and “How do you rate your oral health?” respectively, with five responses: ‘very good’, ‘good’, ‘fair’, ‘poor’, and ‘very poor’. This corresponds to the wording used by Eurostat^33^ with ‘very good’ as the highest ranking. In line with previous studies^6,7^, we constructed binary outcomes separately for oral and general health. We combined ‘very good’ and ‘good’ into *good,* and ‘fair’, ‘poor’, and ‘very poor’ into *poor*^6^.

### Confounders

The centrality index reflects a municipality’s degree of centrality and is based on the population’s commuting time to workplaces and high-order service functions^34^. The centrality index has shown relevance regarding access to medical and dental services. Living in the more central municipalities increases the probability for visits at the dentist, as well as for receiving reimbursements for dental treatment^35^. Hence, people living in less central municipalities visit the dentist (or other health services) less frequently. Based on Statistic Norway’s report, the municipalities were grouped as *least central*, *less central*, *quite central* and *most central*^34^.

Ages (available from registry information) were grouped into the categories: 25–44, 45–66, 67–79, and 80 + years. Respondents aged 18–24 years were excluded from this study because most of them have not finished their tertiary education and similar considerations may be relevant regarding their income^8,20^. Marital status was asked by one question with two categories: *married or cohabiting*, and *single*. Sex was registered according to registry information as *male* or *female*.

### Statistical analyses

All statistical analyses were performed using STATA v16. Descriptive statistics of categorical variables were presented as frequency and percentages. Binary outcome variables for general health and oral health (‘0’ as good health, and ‘1’ as poor health) were constructed. The exposure variables were: education (three levels), with higher education as reference group; income level, categorized into quintiles, with Q5 (highest) as reference group, and economic security, with *yes* (able to pay unforeseen expense) as reference group.

The complex survey design and unequal probabilities of sample selection were taken into account using the ‘svyset’ command in Stata to acknowledge inverse probability of selection (pweight) for the sampling weights to generalize our finding to the population in our survey counties, using municipalities as primary sampling units (n = 44), individuals as secondary sampling units and regions (n = 11) as clustering units. Since our outcomes of interest had a prevalence of more than 10%, Prevalence Ratio (PR) as a measure of association was generally suggested rather than Odds Ratio (OR) to avoid overestimation of our study results^36^.

PRs and corresponding 95% confidence intervals were computed to assess the association between socioeconomic determinants and the prevalence of self-reported oral health and general health using Poisson regression models.

Four models were constructed. Model 1 was unadjusted and model 2 was adjusted for age (25–44, 45–66, 67–79, ≥ 80), sex (male, female), marital status (married/living with partner vs single), and mutually adjusted for income level, education level and economic security. Further adjustment for centrality index as a confounder was done in model 3, while model 4 was mutually adjusted for respectively general health (for oral health) and oral health (for general health). Separate analyses for each outcome were performed.

The possible associations between socioeconomic determinants (education level, income level, economic security) and oral health and general health were also evaluated in strata by gender (male vs female) and by age group (< 65 years vs ≥ 65 years). Potential effect modification by gender or age group on the association was assessed by the likelihood ratio test, and a p value of < 0.05 was considered statistically significant.

To check the robustness of our findings, a sensitivity analysis using multilevel Poisson regression analyses, due to the hierarchical structure of the dataset, was performed. Two-level analyses were conducted, with (a) individuals at level 1 using the same set of exposures and potential confounders as in our main analyses, and (b) municipalities at level 2. The intraclass correlation coefficient^37^, a measure of the amount of variation due to a given level, was computed.

### Ethical approval and consent to participate

This study was conducted under license from the Norwegian Data Protection Authority, ref. 14/01453-3/GRA. A Data Protection Impact Assessment (DPIA) was conducted at the Norwegian Institute of Public Health in 2019. Permission to merge survey data with national registry data was obtained from relevant data owners; Statistics Norway, the Norwegian labour and Welfare Administration (NAV) and the Norwegian Tax Administration.

### Consent for publication

The participants were drawn from the National Population Registry and invited through a letter. The letter specified how data should be used, including for research. Consent was given upon participation in the survey.

## Results

Descriptive data are presented in Table 1. The study population included 9068 participants aged ≥ 25 years. The mean age was 55.96 (Standard Deviation 15.55). Women were younger, had attained more education, had lower income level, less possibility of bearing expenses of 10,000 NOK without resorting to loans, and had relatively better oral health than men. The levels of self-reported general health were very similar in men and women.

**Table 1:** Baseline description of the study sample.

	Total (n = 9068)	Male (n = 4290)	Female (n = 4778)
**Age categories, years**			
25–44	2334 (25.7)	1027 (24.0)	1307 (27.4)
45–66	4174 (46.0)	2040 (47.6)	2134 (44.7)
67–79	1980 (21.8)	979 (22.8)	1001 (21.0)
≥ 80	580 (6.4)	244 (5.7)	336 (7.0)
**Marital status**			
Married, cohabiting	6770 (74.7)	3360 (78.3)	3410 (71.4)
Single	2203 (24.3)	885 (20.6)	1318 (27.6)
Missing	95 (1.1)	45 (1.1)	50 (1.1)
**Education level**			
Primary school, ≤ 10 years	1388 (15.3)	606 (14.1)	782 (16.4)
High School, 11–13 years	3858 (42.5)	1941 (45.2)	1917 (40.1)
Higher education, ≥ 14 years	3668 (40.4)	1656 (38.6)	2012 (42.1)
Missing	154 (1.7)	87 (2.03)	67 (1.4)
**Personal annual income, quintiles**^**a**^** (1000 NOK)**			
Q1: < 216	1765 (19.5)	354 (8.3)	1411 (29.6)
Q2: 216–277	1765 (19.5)	647 (15.1)	1118 (23.4)
Q3: 277–335	1765 (19.5)	802 (18.7)	963 (20.2)
Q4: 335–417	1765 (19.5)	1004 (23.4)	761 (16.0)
Q5: > 417	1765 (19.5)	1363 (31.8)	402 (8.4)
Missing	229 (2.5)	115 (2.7)	114 (2.4)
**Economic security**			
Yes	7595 (83.8)	3699 (86.2)	3896 (81.5)
No	1318 (14.5)	531 (12.4)	787 (16.5)
Missing	155 (1.7)	60 (1.4)	95 (2.0)
**Centrality**^**b**^			
Centrality 1, most central	939 (10.4)	431 (10.1)	508 (10.6)
Centrality 2, quite central	5977 (65.9)	2821 (65.8)	3156 (66.1)
Centrality 3, less central	1599 (17.6)	764 (17.8)	835 (17.5)
Centrality 4, least central	553 (61)	274 (6.4)	279 (5.8)
**Self-reported oral health**			
Very good	2268 (25.0)	908 (21.2)	1360 (28.5)
Good	4500 (49.6)	2179 (50.8)	2321 (48.6)
Fair	1500 (16.5)	781 (18.2)	719 (15.1)
Poor	555 (6.1)	304 (7.1)	251 (5.3)
Very poor	152 (1.7)	80 (1.9)	72 (1.5)
Missing	93 (1.0)	38 (0.9)	55 (1.2)
**Self-reported general health**			
Very good	2318 (25.6)	1056 (24.6)	1262 (26.4)
Good	4388 (48.4)	2115 (49.3)	2273 (47.6)
Fair	1542 (17.0)	719 (16.8)	823 (17.2)
Poor	644 (7.1)	320 (7.5)	324 (6.8)
Very poor	81 (0.9)	39 (0.9)	42 (0.9)
Missing	95 (1.1)	41 (0.1)	54 (1.1)

^a^14 cases were incorrectly recorded, therefore they were excluded from the analysis for variable ‘income’.

^b^Centrality (1–4) is influenced by travel time to work and the availability of service features.

Table 2 represents the distribution of socioeconomic determinants in relation to oral and general health. We observed that a higher proportion of individuals with less education reported poor oral or general health than those with more education. Similarly, a considerably higher proportion of individuals with poor oral and general health were found in the lowest quintile (Q1) of the income level than in the highest quintile (Q5). Furthermore, individuals who could afford to pay 10,000 NOK without resorting to loans reported considerably better oral and general health than those who could not.

**Table 2:** Distribution of self-reported “poor general health” and “poor oral health” by level of education, personal income and economic security.

	Oral health^a^	General health^a^
	N (%) with poor health	N (%) with poor health
**Education level**		
Primary school ≤ 10 years	482 (22.3)	547 (24.5)
High school 11–13 years	1047 (48.5)	1088 (48.7)
Higher education ≥ 14 years	630 (29.2)	597 (26.7)
	2159 (100)	2232 (100)
**Personal annual income level, quintiles (1000 NOK)**		
Q1: < 216	552 (25.8)	646 (29.0)
Q2: 216–277	522 (24.4)	595 (26.7)
Q3: 277–335	425 (19.9)	432 (19.4)
Q4: 335–417	343 (16.1)	320 (14.4)
Q5: > 417	295 (13.8)	232 (10.4)
	2137 (100.0)	2225 (100.0)
**Economic security**		
Yes	1600 (74.0)	1690 (76.4)
No	562 (26.0)	522 (23.6)
	2162 (100.0)	2212 (100.0)

^a^‘Poor’ is defined as those who self-reported general health or oral health as fair, poor and very poor.

Table 3 shows the results of association between socioeconomic factors and self-reported oral health and general health as outcomes. Model 1 was unadjusted. In model 2, adjusted for age, sex, marital status, income level, and economic security, those with primary education were 1.43 times and 1.54 times more likely to report poor oral and general health, respectively, than the highest educational group. Regarding income, individuals within the lowest quintile (Q1) were 1.60 and 2.35 times more likely to report poor oral health and general health, respectively, than the highest income quintile (Q5). Further, individuals who could not afford to pay the sum of 10,000 NOK without resorting to loans were 1.88 times more likely to report poor oral health, and 1.62 times more likely to report poor general health, than those who could afford to pay. Further adjustment for the centrality variable in model 3 did not change the PRs for poor oral and general health. Model 4 includes all the variables in model 3 with mutual adjustments for the confounders self-reported oral health and general health status. In this model, the associations between the three socioeconomic determinants and the outcomes were slightly attenuated, while the gradients remained significant. In model 4, PR for those with primary education was 1.27 for poor oral health and 1.43 for poor general health. Correspondingly, the PR for the lowest income quintile was 1.34 for poor oral health and 2.10 for poor general health. Similarly, in the adjusted model 4, those who could not afford to pay an unexpected bill were 1.65 and 1.37 times more likely to have poor self-reported oral health and general health, respectively, than those who could afford to pay.

**Table 3:** Prevalence Ratio of socioeconomic determinants in relation to self-reported oral and general health.

Variables	Oral health				General health			
	Model 1, PR (95% CI)	Model 2, PR (95% CI)	Model 3, PR (95% CI)	Model 4, PR (95%CI)	Model 1, PR (95% CI)	Model 2, PR (95% CI)	Model 3, PR (95% CI)	Model 4, PR (95% CI)
	N = 8825	N = 8495	N = 8495	N = 8454	N = 8822	N = 8486	N = 8486	N = 8454
**Education level**								
Primary school ≤ 10 years	2.00 (1.81–2.23)	1.43 (1.27–1.62)	1.43 (1.26–1.61)	1.27 (1.11–1.46)	2.45 (2.16–2.77)	1.54 (1.39–1.70)	1.53 (1.39–1.69)	1.43 (1.29–1.59)
High school 11–13 years	1.57 (1.43–1.71)	1.32 (1.23–1.42)	1.32 (1.23–1.42)	1.24 (1.16–1.33)	1.74 (1.53–1.97)	1.32 (1.19–1.47)	1.32 (1.19–1.46)	1.27 (1.15–1.40)
Higher education ≥ 14 years	1.00 (ref)	1.00 (ref)	1.00 (ref)	1.00 (ref)	1.00 (ref)	1.00 (ref)	1.00 (ref)	1.00 (ref)
p-linear trend	**< 0.001**	** < 0.001**	** < 0.001**	** < 0.001**	** < 0.001**	** < 0.001**	** < 0.001**	** < 0.001**
Per gradient increase^a^	1.42 (1.35–1.50)	1.20 (1.13–1.28)	1.20 (1.13–1.28)	1.14 (1.06–1.21)	1.57 (1.47–1.67)	1.24 (1.18–1.31)	1.24 (1.18–1.30)	1.19 (1.16–1.22)

	N = 8738	N = 8495	N = 8495	N = 8454	N = 8732	N = 8486	N = 8486	N = 8454
**Personal annual income, quintiles**								
Q1 (lowest)	1.83 (1.56–2.14)	1.60 (1.39–1.85)	1.60 (1.39–1.83)	1.34 (1.17–1.55)	2.78 (2.42–3.19)	2.35 (2.03–2.72)	2.34 (2.02–2.71)	2.10 (1.82–2.43)
Q2	1.73 (1.50–2.00)	1.51 (1.30–1.75)	1.50 (1.30–1.74)	1.29 (1.11–1.49)	2.56 (2.25–2.91)	2.15 (1.86–2.48)	2.14 (1.86–2.47)	1.95 (1.69–2.23)
Q3	1.45 (1.26–1.68)	1.33 (1.16–1.52)	1.32 (1.16–1.51)	1.23 (1.08–1.39)	1.84 (1.56–2.17)	1.69 (1.44–1.98)	1.68 (1.43–1.98)	1.58 (1.34–1.85)
Q4	1.14 (0.95–1.37)	1.14 (0.96–1.35)	1.14 (0.96–1.35)	1.09 (0.93–1.28)	1.39 (1.15–1.68)	1.39 (1.17–1.66)	1.39 (1.17–1.65)	1.35 (1.14–1.59)
Q5 (highest)	1.00 (ref)	1.00 (ref)	1.00 (ref)	1.00 (ref)	1.00 (ref)	1.00 (ref)	1.00 (ref)	1.00 (ref)
p-linear trend	**< 0.001**	** < 0.001**	** < 0.001**	** < 0.001**	** < 0.001**	** < 0.001**	** < 0.001**	** < 0.001**
Per gradient increase^b^	1.18 (1.14–1.21)	1.13 (1.10–1.16)	1.13 (1.10–1.16)	1.08 (1.05–1.11)	1.29 (1.26–1.32)	1.23 (1.20–1.26)	1.23 (1.20–1.26)	1.20 (1.14–1.26)

	N = 8848	N = 8495	N = 8495	N = 8454	N = 8968	N = 8486	N = 8486	N = 8454
**Economic security**								
No	2.04 (1.91–2.19)	1.88 (1.74–2.02)	1.88 (1.72–2.05)	1.65 (1.53–1.78)	1.80 (1.63–1.99)	1.62 (1.46–1.78)	1.61 (1.46–1.78)	1.37 (1.25–1.51)
Yes	1.00 (ref)	1.00 (ref)	1.00 (ref)	1.00 (ref)	1.00 (ref)	1.00 (ref)	1.00 (ref)	1.00 (ref)

Model 1 was unadjusted.

Model 2 was adjusted for age (four categories), sex, marital status (single, cohabitant/married) and mutually adjusted for education, income, economic security.

Model 3 includes variables in Model 2 plus centrality (in four categories).

Model 4 includes variables in Model 3 and was mutually adjusted for self-reported oral health status and general health status.

^a^PR for per gradient increase in education.

^b^PR for per gradient increase in income.

Significant values are in bold.

Overall, we observed positive linear trends between education level and oral and general health (P_linear trend_ < 0.001 for both outcomes). Similar trends were observed regarding income level. The PR for each gradient increase of income was higher for general health (PRinc, 1.20, 95%CI, 1.14–1.26) than for oral health (PRinc, 1.08, 95%CI, 1.05–1.11), and the educational gradients for oral and general health were quite similar.

### Effect modification by age group and gender to oral health and general health

The level of education was considerably associated with oral health among those aged below 65 years, the common retirement age in Norway, whereas the association was relatively weaker among those aged equal to or over 65 years. The likelihood ratio test showed significant effect modification by the age group (p = 0.032). Likewise, we also observed considerable association with level of education and general health in both < 65 years and ≥ 65 years age groups. However, the point estimates for primary school education were relatively larger in those aged < 65 years than ≥ 65 years. The likelihood ratio test showed significant effect modification by age group (p = 0.021). Further, we found no evidence of effect modification by age group between income level and oral health and general health (See Supplementary Table 1).

In Supplementary Table 2, we further examined if the association between education level, income level and both outcomes was modified by gender. We found no statistical evidence of effect modification by gender between education level and oral health (p = 0.111) and general health (p = 0.259). However, we found statistical evidence of effect modification by gender between income levels for oral health (p = 0.0035), but not for general health.

Our sensitivity analysis suggests that results did not change from the main analyses when the multilevel Poisson regression approach was applied (see Supplementary Table 3). In the multilevel analysis (random-effect parameters), the intercept and intra-class correlation coefficients^37^ for municipalities were slightly different from zero in all the models, suggesting that the PR varied only slightly between municipalities, indicating that multilevel modelling was not required.

## Discussion

Using data from a large cross-sectional Norwegian study, our objective was to investigate the association between socioeconomic determinants and self-reported oral and general health. This study demonstrated that lower levels of education, income, or lack of economic security were associated with an increased likelihood of reporting poor oral and general health in the adjusted model. Further, the results did not change after adjustment for the centrality index. However, the prevalence estimates were slightly attenuated after further mutual adjustments for both oral and general health status. Our findings suggesting similar educational gradients in both oral and general health are in line with our proposed hypothesis. Further, we expected more substantial differences in oral health than in general health according to income level, based on our present Norwegian social welfare system. However, the income gradient was found to be more pronounced for general health than for oral health, which was the opposite of what we hypothesized. Furthermore, using a likelihood ratio test, we found evidence of effect modification by gender between income level and oral health, and by age group between education level and general health.

In line with our findings, a cross-sectional study from the United States (NHANES phase III, 1988–1994) including participants 17 years or older, suggested clear income and education gradients in relation to both oral and general health, indicating that the same social determinants may be involved in both outcomes^4^. Another large cross-sectional study from the following NHANES Surveys (1999–2014) also suggested that higher levels of education and income were associated with higher odds of reporting excellent or very good oral and general health. This study also demonstrated that self-reported general health was significantly associated with self-reported oral health, and this was in line with our findings^3^. Furthermore, Borrell and Baquero’s study from the United States also reported higher levels of education and income to be positively associated with self-reported oral and general health. In addition, they calculated a composite neighbourhood socioeconomic score, and found no association between this score and any of the health outcomes^38^. However, this score was not comparable to our centrality index.

The investigation led by Hakeberg and Boman^7^ was conducted in a similar setting and reported findings in line with our study, including a higher gradient in ORs for poor general health according to income level compared to the corresponding gradient for oral health. Hakeberg and Boman also reported positive associations between economic security and oral and general health, while the magnitude of effect measures regarding general health was slightly higher compared to our study. This could be explained by different categorisation of the economic security variable, which might have led to underestimation of our study findings.

Generally, the health system in Norway covers all major expenses for somatic and mental health care except oral care, while only a few selected oral treatments are covered for small groups of the population. The Swedish general health care system is similar to the Norwegian system, while the Swedish oral care insurance scheme ensures that unexpectedly high oral treatment costs are reduced^26^. Economic security seems to be an important indicator for measuring socioeconomic inequalities in both oral health and general health in both countries, and may encompass another socioeconomic dimension of poverty than the lowest quintiles of income^39^. The variable *economic security* may therefore be of interest when comparing differences between oral and general health. In our study, lack of economic security was associated with poor oral and general health, and this association was significantly stronger for oral health than for general health (see model 4 in Table 3). However, our hypothesis of a more pronounced association between income and oral health than between income and general health was not supported. Instead the association was found to be stronger for general than for oral health. This may partially be explained by the unlimited, free dental services in childhood and youth that provide Norwegians with a good foundation for good dental health later in life, irrespective of income. Other important factors that may influence people’s oral health could be raised awareness regarding maintaining good oral hygiene, low sugar intake and the perceived importance of oral health.

Recent results from the Norwegian part of the EU-SILC surveys showed that the most important reason for unmet needs for dental care was personal economy^29^. Though dental care for adults in Norway in general is private, it is possible to receive disbursements for specific kinds of care. Grytten (2021) notes that even if this is theoretically equally available to all, people with more education are more likely to receive such disbursements^40^. Thus, income does not seem to be more important than education.

Surprisingly, and in contrast to our study, Hadler-Olsen and Jönsson^11^, who studied self-reported oral health and the use of oral health services in the adult population in Northern Norway did not find education level to be significantly associated with self-reported oral health. This discrepancy may be influenced by their relatively smaller sample size, and possibly the use of education variables based on questionnaire rather than registry data, as in our study. Especially young adults faced financial barriers against receiving dental health services and also had poorer self-reported oral health^11^. In our study, the PR for self-reported poor oral health was higher (PR = 1.65) in the population group which lacked economic security compared to the group with more savings.

The overall sex differences in our study were found to be small, but the prevalence ratio for poor oral health in the lowest income quintile (Q1) was higher in women (PR 2.97) than in men (PR 1.40), indicating that regarding oral health, women may be more vulnerable than men to having low income, possibly contributing to difficulties in consulting dental care^35^ (Supplementary Table 1). This is in contrast to Maldi et al., who reported time trends in income and educational inequalities using three waves of cross-sectional data and found marked sex differences, including more fluctuating trends in self-reported (general) health outcomes for women than for men^8^. A possible explanation might be that the socioeconomic positions of men and women in rural Northern Sweden in 2006, 2010 and 2014 were not fully comparable to the socioeconomic position of men and women in the more urban Southern Norway in 2015–2016.

Overall, we found a similar socioeconomic distribution for self-reported oral and general health. The relatively generous social benefits for those in the lowest income groups in Norway may partly compensate for the dental treatment costs^35^. Another reason for the similarity may be the mutual influence of health problems between two outcomes, in that poor general health will influence oral health negatively, and vice versa. Even though there was strong bidirectional association between oral health and general health in our study (data not shown), the gradients remained significant for income and education level in all models, also when mutually adjusting for oral and general health. Interestingly, the observed differences clearly show a large potential to improve and reduce socio-economic disparities in oral and general health in Norway. The most obvious remedy is to improve the availability of health services for all population groups, with special focus on those with low income and education^11,23,24^. Early studies of different aspects of self-reported oral health found self-reported general health to be a significant predictor of most oral health measures, such as oral pain or number of teeth^24^. Similarly, income and education gradients in oral and general health were reported by Sabbah, implying commonalities of the social determinants of both measures. Li et al. found self-reported general health to be significantly associated with self-reported oral health after adjusting for other sociodemographic factors^3,41^. In our data, the economic differences seemed to have a larger impact on general health than on oral health. Economic security, however, was more strongly associated with oral health than with general health. The strong bidirectional association between our two outcomes may also be an argument for the integration of dental health services and general health services, at least regarding financial reimbursements. Beyond the scope of this paper, additional questions about dental visits, dental hygiene, and postponed dental visits for financial reasons could have given opportunities to investigate other dimensions of socioeconomic differences in oral health.

Though oral health can be seen as an individual phenomenon, it is nevertheless important to study and discuss oral health on a societal level^42^. Even in wealthy countries like Norway, social conditions influence individuals’ choices, which are limited by nutrition policy, price levels and cultural traditions^43^. Education can be seen as a proxy for many individual lifestyle factors^44^. Most of the recommended measures to combat socioeconomic inequalities in health are directed towards childhood, upbringing and education, but also advocate strengthened measures for smoking cessation and taxation of sweetened beverages^2,43^. Income and wealth may act as the direct, main determinants of health inequalities, but the influencing factors are also linked to position and social structure^25^. Long-term economic and social stress are believed to affect biological processes that can increase the predisposition to disease. Increased financial stress is found to be related to increased levels of stress hormones and cariogenic bacterial counts in dental caries^45^. Similar mechanisms may affect other disease courses as well. A better integration of dentistry with primary care services may offer opportunities to reduce the social inequality gap in oral health^1,2,43^.

Our study has contributed with new knowledge in different ways. Three exposures were used to measure different dimensions of socioeconomic inequality; education, income, and economic security. This study contributes to updating knowledge about the state of socioeconomic disparities in self-reported oral and general health in the Nordic countries, as we found few studies from this area^9,17,19,20^, and especially few recent studies^7,8,11^.

Our study has several strengths. First, we had adequate power to draw statistical inference from our study findings. Second, the sample was randomly drawn from the municipalities of three large counties, representing a large proportion of the Norwegian population^30,31^. Third, the data on education and personal income were obtained from the national population-based registers of Statistics Norway, which largely reduces the possibility of information bias and ensures available data for the large majority of participants. Fourth, our study examined the contribution of personal income rather than family income. In Norway, where the overwhelming majority of women are in paid work^46^, personal income may be a better indicator than family income. Fifth, for our outcomes, we used validated questions, corresponding with comparable objective variables^12,13,15,24^. Sixth, we included a confounder, the *centrality index*, to assess the association between socioeconomic determinants and oral and general health, which—to our best knowledge—previously no study had included.

Our study also has several limitations. First, using self-reported questionnaires might have led to recall bias. Second, due to the cross-sectional nature of the study, the issue of reverse causality cannot be ruled out. Third, the issue of residual confounding cannot be ignored because of unmeasured, mismeasured or misspecified variables. Fourth, there might be a certain degree of selection bias in the direction of overrepresentation of middle aged, women and highly educated^30^. Fifth, information about the potential mediators, such as use of dental services and time since the last visit was not available. Hence, future studies should assess the mediating effect of use of health services and time since the last visit between socioeconomic status and oral and general health.

## Conclusions

This study fills a gap of knowledge, as few recent studies of self-reported oral and general health have been carried out in the Nordic countries. Self-reported oral and general health were associated with educational level, income level and economic security in a pattern of gradients with positive linear trends. Including oral health conditions along with other somatic and mental diseases within the health care system may contribute to improving both oral and general health among people in low socioeconomic groups.

## Supplementary Information

Supplementary Tables.

## Data Availability

Anonymised data used in this study may be available upon request from the Norwegian Institute of Public Health (NIPH) and after permission from the county councils of Vestfold, Aust-Agder and Vest-Agder. Extra restrictions apply to the availability of the data set used in this article linked to variables from national registries. This requires permission from the registry owners and the Norwegian Data Protection Authority.
