# Supplementary material for: A study of socio-economic inequalities in self-reported oral and general health in South-East Norway
Source: Sci Rep. 2022 Aug 12;12:13721. doi: 10.1038/s41598-022-18055-5 (PMC9374767; doi:10.1038/s41598-022-18055-5)
Supplement: Supplementary file 1 — Supplementary Tables. [file 41598_2022_18055_MOESM1_ESM.docx]

### Supplementary Table 1. Analyses of socioeconomic determinants in relation to self-reported oral and general health, stratified for males and females^1,2^

|  | **Oral Health**^3^ | | | **General Health**^3^ | |
| --- | --- | --- | --- | --- | --- |
|  | **Male**  **PR (95% CI)**  **(n=4021)** | **Female**  **PR (95% CI)**  **(n=4474)** | | **Male**  **PR (95% CI)**  **(n=4016)** | **Female**  **PR (95% CI)**  (n=4470) |
| **Education level** |  |  |  | |  |
| Primary school ≤ 10 years | 1.34 (1.09-1.37) | 1.48 (1.18-1.86) | 1.40 (1.20-1.63) | | 1.63 (1.40-1.90) |
| High school 11-13 years | 1.22 (1.09-1.37) | 1.41 (1.18-1.68) | 1.25 (1.09-1.43) | | 1.37 (1.20-1.56) |
| Higher education ≥ 14 years | 1.00 (ref.) | 1.00 (ref.) | 1.00 (ref.) | | 1.00 (ref.) |
|  | *0.111* |  | *0.259* | |  |
| **Personal annual income, quintiles** |  |  | |  |  |
| Q1 (Lowest) | 1.40 (1.17-1.69) | 2.97 (1.76-4.99) | | 2.21 (1.90-2.57) | 2.36 (1.62-3.44) |
| Q2 | 1.46 (1.22-1.76) | 2.65 (1.56-4.50) | | 2.26 (1.86-2.73) | 2.11 (1.43-3.10) |
| Q3 | 1.23 (1.03-1.48) | 2.43 (1.53-3.87) | | 1.76 (1.51-2.04) | 1.64 (1.10-2.47) |
| Q4 | 1.07 (0.87-1.31) | 2.04 (1.20-3.45) | | 1.49 (1.23-1.80) | 1.26 (0.84-1.88) |
| Q5 (Highest) | 1.00 (ref.) | 1.00 (ref.) | | 1.00 (ref.) | 1.00 (ref.) |
|  | ***0.0035*** |  | | *0.565* |  |
|  |  |  | |  |  |

^1^ Prevalence ratio

^2^ Models were adjusted for age categories, marital status and centrality

^3^ Binary outcome for general health and oral health: ‘0’ as good health, and ‘1’ as poor health

### Supplementary Table 2. Analyses of socioeconomic determinants in relation to self-reported oral and general health, stratified for two age group^1,2^

|  | **Oral Health**^3^ | | **General Health**^3^ | |
| --- | --- | --- | --- | --- |
|  | **Age < 65**  **PR (95% CI)** | **Age ≥ 65**  **PR (95% CI)** | **Age < 65**  **PR (95% CI)** | **Age ≥ 65**  **PR (95% CI)** |
| **Education level** |  |  |  |  |
| Primary school ≤ 10 years | 1.66 (1.36-2.03) | 1.11 (0.95-1.30) | 1.68 (1.50-1.87) | 1.34 (1.10-1.63) |
| High school 11-13 years | 1.35 (1.15-1.58) | 1.06 (0.93-1.21) | 1.30 (1.15-1.46) | 1.31 (1.07-1.60) |
| Higher education ≥ 14 years | 1.00 (ref) | 1.00 (ref) | 1.00 (ref) | 1.00 (ref) |
| *Likelihood ratio test* |  | ***0.032*** | ***0.021*** |  |
| **Personal annual income, quintiles** | |  |  |  |
| Q1 (Lowest) | 1.65 (1.42-1.93) | 1.52 (1.10-2.08) | 2.46 (1.16-2.80) | 1.10 (0.80-1.51) |
| Q2 | 1.47 (1.22-1.77) | 1.49 (1.14-1.94) | 2.24 (1.95-2.57) | 1.44 (1.11-1.87) |
| Q3 | 1.38 (1.17-1.63) | 1.20 (0.86-1.67) | 1.77 (1.51-2.08) | 1.84 (1.34-2.51) |
| Q4 | 1.24 (1.03-1.49) | 0.89 (0.61-1.31) | 1.52 (1.28-1.80) | 2.00 (1.41-2.84) |
| Q5 (Highest) | 1.00 (ref) | 1.00 (ref) | 1.00 (ref) | 1.00 (ref) |
| *Likelihood ratio test* | *0.505* | | *0.825* | |
| **Economic security** |  |  |  |  |
| No | 1.99 (1.81-1.19) | 1.64 (1.44-1.86) | 1.68 (1.49-1.89) | 1.45 (1.26-1.67) |
| Yes | 1.00 (ref) | 1.00 (ref) | 1.00 (ref) | 1.00 (ref) |
| ***N*** | 5721 | *0.203*  2774 | *0.301*  5720 | 2766 |

^1^ Prevalence ratio

^2^ Models were adjusted for sex, marital status and centrality

^3^ Binary outcome for general health and oral health: ‘0’ as good health, and ‘1’ as poor health

### Supplementary Table 3 Multilevel analyses between socioeconomic determinants and self-reported oral and general health^1,2^

|  | **Oral health** | | | **General health** | | |
| --- | --- | --- | --- | --- | --- | --- |
|  | **Model 1**  **OR (95% CI)** | **Model 2**  **OR (95% CI)** | **Model 3**  **OR (95% CI)** | **Model 1**  **OR (95% CI)** | **Model 2**  **OR (95% CI)** | **Model 3**  **OR (95% CI)** |
| Fixed effect |  |  |  |  |  |  |
| Intercept^3^ | -1.379 (0.030) | -1.90 (0.115) | 0.96 (0.110) | -1.346 (0.029) | -2.472 (0.113) | -2.616 (0.098) |
|  |  |  |  |  |  |  |
| **Individual-level factors** |  |  |  |  |  |  |
| Education level |  |  |  |  |  |  |
| Primary school ≤ 10 years |  | 1.43 (1.27-1.61) | 1.28 (1.13-1.45) |  | 1.51 (1.37-1.66) | 1.41 (1.17-1.57) |
| High school 11-13 years |  | 1.32 (1.23-1.41) | 1.24 (1.17-1.32) |  | 1.31 (1.18-1.45) | 1.26 (1.14-1.39) |
| Higher education ≥ 14 years |  | 1.00 (ref) | 1.00 (ref) |  | 1.00 (ref) | 1.00 (ref) |
|  |  |  |  |  |  |  |
| Personal annual income,  quintiles |  |  |  |  |  |  |
| Q1 (Lowest) |  | 1.60 (1.40-1.83) | 1.35 (1.18-1.54) |  | 2.34 (2.03-2.70) | 2.10 (1.82-2.42) |
| Q2 |  | 1.49 (1.29-1.73) | 1.28 (1.11-1.48) |  | 2.14 (1.86-2.46) | 1.95 (1.70-2.23) |
| Q3 |  | 1.32 (1.16-1.50) | 1.22 (1.08-1.38) |  | 1.68 (1.43-1.96) | 1.57 (1.34-1.84) |
| Q4 |  | 1.13 (0.96-1.33) | 1.09 (0.93-1.27) |  | 1.38 (1.17-1.64) | 1.35 (1.15-1.58) |
| Q5 (Highest) |  | 1.00 (ref) | 1.00 (ref) |  | 1.00 (ref) | 1.00 (ref) |
|  |  |  |  |  |  |  |
| Economic security |  |  |  |  |  |  |
| No |  | 1.88 (1.75-2.02) | 1.65 (1.54-1.78) |  | 1.61 (1.46-1.78) | 1.37 (1.25-1.50) |
| Yes |  | 1.00 (ref) | 1.00 (ref) |  | 1.00 (ref) | 1.00 (ref) |
|  |  |  |  |  |  |  |
| Gender |  |  |  |  |  |  |
| Male |  | 1.00 (ref) | 1.00 (ref) |  | 1.00 (ref) | 1.00 (ref) |
| Female |  | 0.69 (0.62-0.73) | 0.71 (0.65-0.76) |  | 0.78 (0.74-0.82) | 0.85 (0.81-0.90) |
|  |  |  |  |  |  |  |
| Age (categories) |  |  |  |  |  |  |
| 25-44 |  | 1.00 (ref) | 1.00 (ref) |  | 1.00 (ref) | 1.00 (ref) |
| 45-66 |  | 1.04 (0.94-1.15) | 0.94 (0.85-1.04) |  | 1.56 (1.42-1.73) | 1.53 (1.39-1.69) |
| 67-79 |  | 1.28 (1.18-1.39) | 1.15 (1.05-1.25) |  | 1.68 (1.53-1.85) | 1.59 (1.45-1.75) |
| 80+ |  | 1.27 (1.05-1.53) | 1.09 (0.91-1.32) |  | 1.94 (1.67-2.25) | 1.82 (1.54-2.14) |
| Marital Status |  |  |  |  |  |  |
| Cohabitant/married |  | 1.00 (ref) | 1.00 (ref) |  | 1.00 (ref) | 1.00 (ref) |
| Single |  | 1.25 (1.14-1.38) | 1.19 (1.09-1.30) |  | 1.22 (1.13-1.31) | 1.15 (1.06-1.24) |
| Centrality |  |  |  |  |  |  |
| 1 |  | 1.00 (ref) | 1.00 (ref) |  | 1.00 (ref) | 1.00 (ref) |
| 2 |  | 0.87 (0.73-1.04) | 0.88 (0.74-1.05) |  | 0.94 (0.84-1.06) | 0.97 (0.89-1.06) |
| 3 |  | 1.02 (0.84-1.23) | 1.00 (0.83-1.19) |  | 1.03 (0.88-1.19) | 1.02 (0.91-1.14) |
| 4 |  | 1.02 (0.81-1.30) | 1.01 (0.81-1.25) |  | 1.01 (0.84-1.21) | 0.98 (0.84-1.14) |
| **Random-effect parameters** |  |  |  |  |  |  |
| Lower level (individuals) | 8975 | 8495 | 8589 | 8973 | 8486 | 8454 |
| Higher level (municipalities) | 44 | 44 | 44 | 44 | 44 | 44 |
| Variance, σ^2^_u_ (SE) | 0.011(0.007) | 0.030 (0.009) | 0.025 (0.006) | 0.008(0.005) | 0.149 (0.017) | 0.035 (0.009) |
| ICC (%) | 0.89% | 2.57% | 2.43% | 0.71% | 2.0% | 1.98% |
| ***Model fit statistics*** |  |  |  |  |  |  |
| AIC | 10603.14 | 9557.412 | 9237.152 | 10770.61 | 9654.902 | 9350.315 |
| BIC | 10617.34 | 9677.215 | 9363.916 | 10784.82 | 9767.641 | 9470.035 |

^1^ Odds ratio

^2^ Model 2 was adjusted for (age categories), sex and marital status. Model 3 includes variables in Model 2 plus centrality

^3^Intercept is presented as coefficient (standard error)

SE: Standard Error. ICC: Intra class correlation. AIC=Akaike information criterion, BIC = Bayesian information criterion. The value of AIC and SBIC were used as model fit statistics.
